# Higher early proximal migration of hemispherical cups with electrochemically applied hydroxyapatite (BoneMaster) on a porous surface compared with porous surface alone: a randomized RSA study with 53 patients

**DOI:** 10.1080/17453674.2019.1687860

**Published:** 2019-11-08

**Authors:** Peter Bo Jørgensen, Henrik Daugaard, Stig Storgaard Jakobsen, Martin Lamm, Kjeld Søballe, Maiken Stilling

**Affiliations:** a Department of Orthopedic Surgery, Aarhus University Hospital, Aarhus;; b Department of Clinical Medicine, Aarhus University, Aarhus;; c Department of Orthopaedic Surgery, Gentofte Hospital, Hellerup, Denmark

## Abstract

Background and purpose — BoneMaster (BM) is an electrochemically deposited hydroxyapatite (HA) implant-coating, which is evenly distributed, thin, and quickly resorbed. It is designed to stimulate osseointegration and early implant stability and alleviate longer-term HA-induced third-body polyethylene wear. This study evaluates early cup migration and functional outcomes of cementless porous-coated hemispherical cups with or without BM.

Patients and methods — In a patient-blinded, randomized, controlled trial 53 patients at mean 64 years (55–75) with coxarthritis were operated with an Exceed cup (Zimmer Biomet) and Bi-Metric stem (Zimmer Biomet) with porous and BM coating (PBM) or with porous coating alone (P). Follow-ups were performed postoperatively and at 3, 6, 12, and 24 months. Effect measures were cup migration measured with RSA and PROMs.

Results — At 6-month follow-up, proximal cup migration in the PBM group (0.09 mm, 95% CI 0.02–0.20) was higher than in the P group (0.25 mm, CI 0.15–0.35). At 1- and 2-year follow-up, cup migration in all 6 degrees of freedom was similar between groups (p > 0.2). From before surgery to 2-year follow-up, Oxford Hip Score (OHS) increased by 17 points (CI 14–20). Hip disability and Osteoarthritis Outcome Score (HOOS) increased in all sub-scores, but was more pronounced for PBM cups compared with P cups in the Symptoms sub-score (p = 0.04).

Interpretation — Contrary to expectations, PBM cups had higher early migration than P cups. At 2-year follow-up, migration was similar between groups. There seems to be no early benefit of BM coating on acetabular cups.

Aseptic loosening remains one of the most common reasons for cup revision. In Denmark up to 86% of cups are cementless and 35% of these are coated with hydroxyapatite (HA) (DHR 2016).

Plasma-sprayed HA coating results in better bony ingrowth and early implant fixation in experimental studies (Soballe et al. 1992, Daugaard et al. 2010). However, clinical results have not truly been able to show superior cementless cup fixation with HA over porous coating at midterm (Rohrl et al. 2004, Valancius et al. 2013) or long-term (Otten et al. 2016, Lazarinis et al. 2017).

Plasma-sprayed HA applies coating in a line-of-sight, which may reduce the microstructure in the porous coating. Electrochemical application of HA is a new technique resulting in a thinner coating layer with better topographic structure and distribution as compared with plasma-sprayed HA. Electrochemically applied HA may stimulate early implant osseointegration and is resorbed within a few months (Daugaard et al. 2010). Therefore, it may not contribute to polyethylene wear.

The osteoconductive properties of electrochemical HA coating have been validated in experimental studies (Wang et al. 2006, Daugaard et al. 2010) and clinically on a femoral stem using RSA (Boe et al. 2011, Flatoy et al. 2016) in accordance with the guidelines for phased introduction (Nelissen et al. 2011). The suggested thresholds for proximal cup migration are 0.2 mm (at risk) and 1.0 mm (unacceptable), and for every 1 mm of proximal migration the risk of later revision increases by 10% (Pijls et al. 2012).

We evaluated the effect of electrochemically applied HA coating on early cup migration and functional outcomes using porous coated Exceed cups (Zimmer Biomet, Warsaw, IN, USA). We hypothesized that cementless Exceed ABT cups with electrochemically applied HA coating would have superior or equal early fixation compared to identical cups without hydroxyapatite coating.

## Patients and methods

### Study design

**Figure 1.**
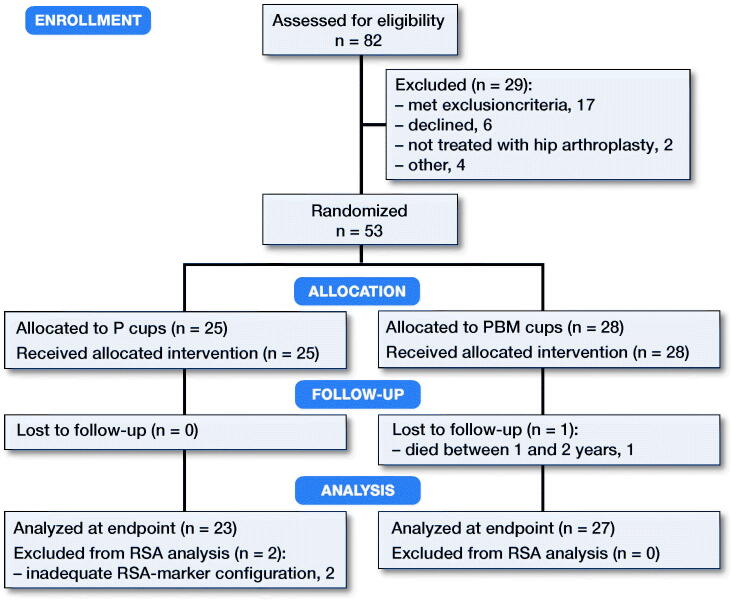
Consort flow chart

In this patient-blinded, randomized controlled trial, 82 patients were assessed for eligibility between January 2013 and March 2015. Randomization was done in the theater within blocks of 10 patients (5 porous [P], and 5 porous with BoneMaster [PBM]) by drawing concealed labels from sequentially numbered closed envelopes. We obtained written consent of 56 patients who met the inclusion criteria; non-osteoporotic by a pre-op dual energy X-ray absorptiometry (DEXA) scan, age 55–75 years, and severe coxarthrosis (Figure 1, Table 1).

**Table 1.** Baseline characteristics, mean (95% CI)

Baseline demographics	P cups	PBM cups
N	25	28
Age	65 (63–67)	64 (62–66)
Men/women	11/14	14/14
T-score	–0.3 (–0.8 to 0.2)	–0.8 (–1.2 to –0.4)
OHS-score	27 (23–30)	22 (19–25)
HOOS		
Pain	48 (43–57)	36 (31–45)
Symptoms	45 (40–55)	28 (28–42)
ADL	57 (46–63)	39 (37–50)
Sport	38 (26–43)	13 (13–26)
QoL	38 (30–42)	22 (17–29)

Criteria of exclusion were: other diseases of affected hip than primary coxarthrosis at time of inclusion, secondary osteoarthritis, neuromuscular or vascular condition in lower extremity, arthroplasties of other lower-extremity joints, BMI at time of inclusion ≥ 35 or < 18.5, rheumatoid and similar arthritis, metabolic bone disease, reduced kidney function, previous treatment of skeleton with radiation therapy, pharmaceuticals that effect calcium–phosphorus metabolism and bone density, alcohol abuse, medication abuse, and psychological instability.

### Sample size

A sample size calculation indicated 23 patients per group based on a clinically relevant difference in migration of 0.6 mm (SD 0.6) with a power of 90% and alpha set to 0.05 (Charnley 1982). To balance postoperative dropout, we aimed for 25 patients per group. To balance for exclusions during the inclusion period, we continued inclusion per block randomization until there were minimum 25 patients in each group, and in total 53 patients in the study.

### Implants

25 patients received a cementless Exceed cup (Exceed ABT RingLoc-x shell) size 50-62 mm, and a cementless Bi-Metric stem (Zimmer Biomet). Cup and stem were treated with plasma-spray porous titanium coating with a porosity of 45% and an average pore size of 250 µm (range, 100–1,000 µm), providing a scratch fit (Lindgren et al. 2018). Another 28 patients received similar porous coated Exceed cups (Exceed ABT RingLoc-x shell) size 50–62 mm and Bi-Metric stem (Zimmer Biomet), both with an additional electrochemically applied HA coating (BoneMaster, Zimmer Biomet). The BoneMaster coating consisted of 70% crystalline HA with a thickness of 5 µm and with a 2.0 Ca/P ratio. The amorphous phase in the coating was primarily amorphous calcium phosphate (ACP) but also β-tricalcium phosphate (TCP). All patients received cobalt-chromium-molybdenum modular femoral heads, size 36 mm (1 patient had a size 32), and an E1 highly cross-linked UHMWPE liner (Zimmer Biomet).

### Surgery

All patients were operated at Aarhus University Hospital, Denmark. Preoperative planning was done with the AGFA OT3000 digital templating software (Agfa-Gevaert NV, Mortsel, Belgium) and calibrated digital radiographs. All procedures used a posterolateral approach and the acetabulum was under-reamed by 1 mm in all patients. During surgery 6–8 1-mm tantalum beads were inserted into the peri-prosthetic pelvic bone. Preoperatively, patients received 1.5 g cefuroxime intravenously as antimicrobial prophylaxis, and 1 dose of tranexamic acid 10 mg/kg IV. Postoperatively patients received 1 dose of tranexamic acid IV and 3 doses of 1.5 g cefuroxime IV within the first 24 hours. Postoperatively, the patients were mobilized with full weight-bearing and walking aids as needed, using a “fast track” protocol.

### Radiostereometric analysis

RSA recordings were performed on a standard RSA system with 2 synchronized ceiling-fixed roentgen tubes (Arco-Ceil/Medira; Santax Medico, Aarhus, Denmark) angled toward each other at 40°. The radiographs were digital (Fuji CR, image size 35 x 43) and stored in DICOM file-format without compression. During the study the RSA equipment was replaced with a newer direct digital dedicated stereo X-ray system, AdoraRSA suite (Nordic X-ray Technique, Aarhus, Denmark) with CXDI-70C wireless detectors (Canon, Tokyo, Japan). X-ray tubes remained at a 40-degree angle and a uniplanar carbon calibration box (Box 24, Medis Specials, Leiden, Netherlands) was used for all recordings.

RSA recordings were obtained by experienced radiographers and analyses were performed by the same blinded investigator (PBJ) using CAD surface implant models (Zimmer Biomet) with Model-Based RSA 4.1 (RSAcore, Leiden, The Netherlands). The maximum rigid body error was set to 0.35 mm and the condition number (CN) at 200. The mean CN was 99.8 (95% CI 89.9–109.6). 4 patients (2 in each group) with CN between 150 and 200 had a sufficient and non-linear bone model as judged by visual evaluation, and were kept in the analyses to maintain power. 1 patient with CN above 200 and 1 patient with only 2 markers were excluded from the RSA data.

2 patients had inadequate marker-configuration; 1 had inferior marker position and the other had only 2 visible markers. These patients were removed from the RSA analysis but contributed with patient-reported outcomes. 2 patients received new liners and femoral heads due to instability (PBM 3 days after primary operation) and recurrent dislocation of the hip (P 16 months after primary operation). These patients continued in the study.

### RSA precision

When accepting condition numbers higher than 150 validation of precision becomes essential (Valstar et al. 2005). Precision calculations were based on double RSA examinations recorded at 6-month follow-up (on both RSA systems) (Table 2).

### Radiographs and DEXA scans

Preoperative DEXA scans of the lumbar spine and dual hip region were performed using the 2 fan-beam GE Lunar iDXA with Encore software version 13 (Minneapolis, MN, USA). Patients with a T-score below –2.5 on hip or lumbar spine were defined as osteoporotic according to WHO criteria and excluded.

Standard anterior-posterior and medio-lateral hip radiographs were recorded postoperatively and at 2-year follow-up. Cup position was measured by 1 experienced hip surgeon (KS) using the known diameter of the femoral head to calibrate and avoid magnification errors. Radiolucent lines of 1 mm or more were described according to DeLee and Charnley (1976).

### Patient-reported and clinical outcomes

Patient-reported outcomes were hip disability and osteoarthritis outcome score (HOOS) and Oxford hip score (OHS). HOOS were recorded in 5 subscales (pain, symptoms, ADL, sport, and quality of life) and have been validated for use in patients receiving total hip arthroplasty (Paulsen et al. 2012). Outcome was evaluated in each subscale giving from 0 (worst) to 100 (best) points. OHS was evaluated on a scale from 0 (worst) to 48 (best) points.

Postoperative complications were recorded at the 2-year follow-up.

### Statistics

The RSA dataset consisted of signed cup migrations along and rotations about the 3 orthogonal axes (x, y, z), total translation (TT = sqrt(Tx^2^ + Ty^2^ + Tz^2^)), total rotation (TR = sqrt(Rx^2^ + Ry^2^ + Rz^2^)) and maximum total point motion (MTPM).

All migration measures were normally distributed as evaluated on q–q plots and hence presented as mean and 95% confidence intervals (CI) and were tested using Student’s t-test. Due to the non-normal nature of summed RSA data (TT, TR, and MTPM) the Mann–Whitney U-test was used for statistical testing of those.

Changes in HOOS and OHS were normally distributed as evaluated on q–q plots and tested using Student’s t-test, 1- and 2-year group comparisons were done with the Mann–Whitney U-test. Sub-analysis of proximal migration was performed comparing patients dichotomized on normal bone quality (T score > –1) and osteopenia (T score < –1).

Statistics were calculated using Stata/IC version 13.0 (StataCorp, College Station, TX, USA) and the significance level was set at 0.05.

### Ethics, registration, funding, and potential conflicts of interest

The study was approved by the Data Protection Agency (1-16-02-175-11) and the Central Denmark Regions Committee on Biomedical Research Ethics (M-20110224) and was performed in agreement with the Helsinki II declaration. The study was registered at ClinicalTrials.gov (NCT02311179). Zimmer Biomet supported the study financially but had no influence on the manuscript or publication. The authors have no conflicts of interest.

## Results

### RSA results

Proximal migration was 0.16 mm (CI 0.02–0.30) higher for PBM coated cups (0.25 mm CI 0.15–0.35) compared with P coated cups (0.09 CI –0.02–0.20) at 6-month follow-up. From 1-year follow-up to 2-year follow-up, the proximal migration was 0.0 mm (CI –0.06 to 0.06) for P-coated cups and 0.0 mm (CI –0.05 to 0.04) for PBM-coated cups. At 2-year follow-up, 8 P-coated cups and 10 PBM-coated cups had a proximal migration exceeding the precision limit of 0.2 mm, and the mean 2-year proximal cup migration was 0.09 mm (CI –0.02 to 0.20) for P-coated cups and 0.2 mm (CI 0.10–0.30) for PBM-coated cups. We found no statistically significant difference in proximal migration between the groups at 1-year follow-up (p = 0.2) and at 2-year follow-up (p = 0.2). At 3-month follow-up, we found a statistically significant difference in y-rotation (p = 0.03).

**Figure 2.**
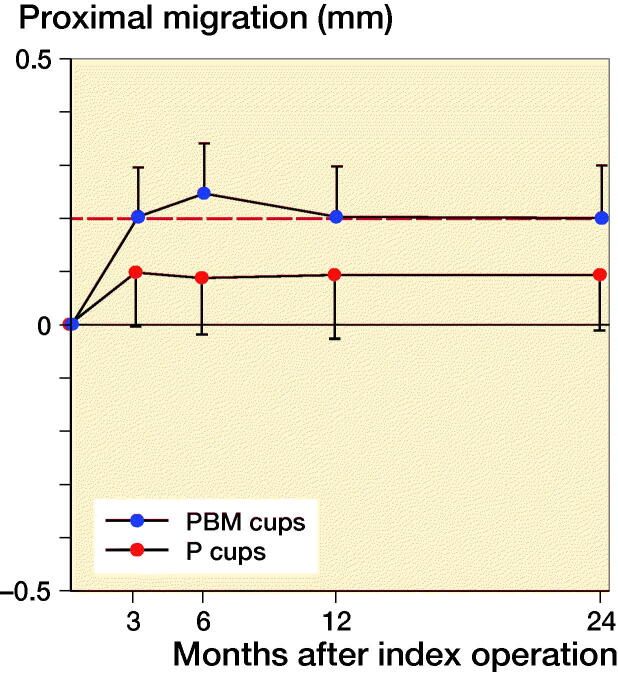
Mean proximal migration (CI) with limit for safe 2-year migration (red line).

**Figure 4.**
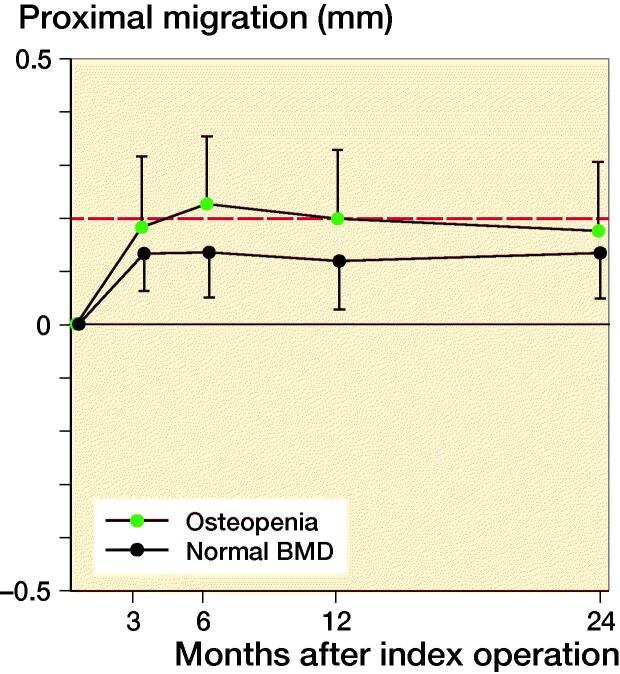
Mean proximal migration (CI) dependent on bone mineral density with limit for safe 2-year migration (red line).

Based on preoperative DEXA scan, 21 patients had osteopenia (T-score < –1) and 29 patients had normal bone quality (T-score > –1). The ratio of normal BMD/osteopenia was 15/8 in the P-coated group and 13/14 in the PBM- coated group. Proximal cup migration was 0.09 mm (CI –0.06 to 0.24) higher for osteopenic patients compared with patients with normal BMD at 6-month follow-up, although this difference was not statistically significant (p = 0.2) (Figure 4). Further RSA results are presented in Figures 2, 3 and 4, and in Tables 2 and 3.

Figure 3.Individual y-migration. Dashed lines are limits of agreement.

**Figure:**
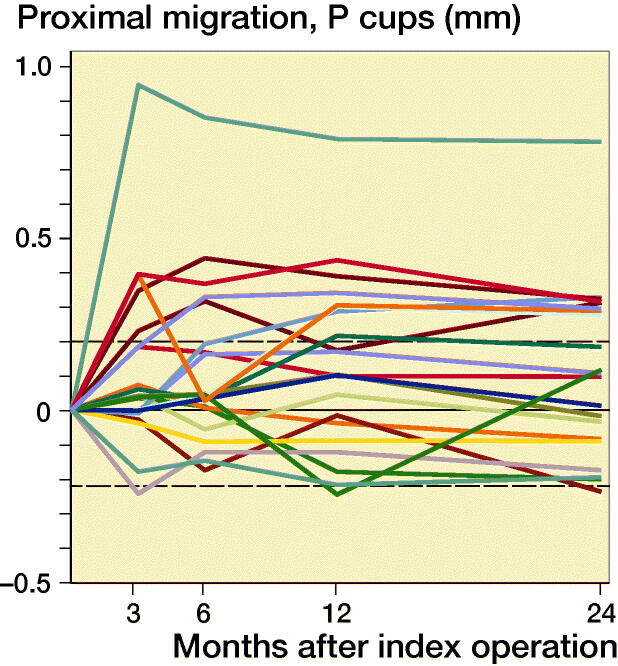

**Figure:**
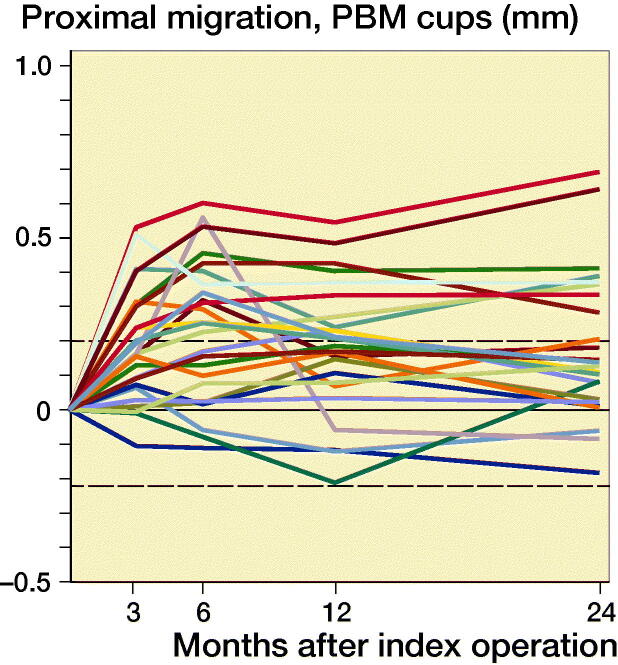

**Table 3.** Migration, mean (95% CI)

	P cups, n = 23	PBM cups, n = 27
Translations, mm		
x-axis (+medial/–lateral)		
3 months	–0.05 (–0.16 to 0.06)	0.01 (–0.14 to 0.16)
6 months	–0.01 (–0.17 to 0.15)	–0.03 (–0.17 to 0.11)
1 year	–0.06 (–0.22 to 0.11)	0.02 (–0.15 to 0.18)
2 years	–0.00 (–0.12 to 0.12)	–0.02 (–0.19 to 0.16)
y-axis (+proximal/–distal)		
3 months	0.10 (–0.01 to 0.21)	0.20 (0.11 to 0.30)
6 months	0.09 (–0.02 to 0.20)	0.25 (0.15 to 0.35) ^a^
1 year	0.09 (–0.03 to 0.22)	0.20 (0.10 to 0.30)
2 years	0.09 (–0.02 to 0.20)	0.20 (0.10 to 0.30)
z-axis (+anterior/–posterior)		
3 months	0.09 (–0.02 to 0.21)	–0.02 (–0.17 to 0.13)
6 months	0.12 (–0.00 to 0.24)	–0.01 (–0.20 to 0.17)
1 year	0.19 (0.05 to 0.33)	0.05 (–0.13 to 0.23)
2 years	0.12 (–0.03 to 0.26)	0.08 (–0.11 to 0.28)
Rotations, degrees		
x-axis (+anterior/–posterior tilt)		
3 months	–0.04 (–0.52 to 0.44)	0.03 (–0.30 to 0.36)
6 months	–0.09 (–0.63 to 0.45)	0.04 (–0.32 to 0.41)
1 year	0.24 (–0.26 to 0.75)	–0.15 (–0.55 to 0.24)
2 years	0.15 (–0.40 to 0.71)	0.02 (–0.35 to 0.40)
y-axis (+internal/–external rotation)		
3 months	–0.32 (–0.82 to 0.18)	0.33 (–0.04 to 0.71) ^a^
6 months	–0.30 (–0.87 to 0.27)	0.23 (–0.24 to 0.70)
1 year	0.14 (–0.55 to 0.82)	–0.12 (–0.63 to 0.39)
2 years	–0.02 (–0.58 to 0.53)	0.27 (–0.28 to 0.82)
z-axis (+increased/–decreased inclination)		
3 months	0.05 (–0.30 to 0.39)	–0.17 (–0.54 to 0.19)
6 months	–0.11 (–0.48 to 0.26)	–0.19 (–0.59 to 0.20)
1 year	–0.32 (–0.78 to 0.14)	–0.13 (–0.57 to 0.32)
2 years	–0.15 (–0.54 to 0.25)	–0.20 (–0.65 to 0.25)
Maximum total point movement ^b^		
3 months	0.94 (0.74 to 1.20)	1.01 (0.85 to 1.20)
6 months	1.05 (0.81 to 1.38)	1.19 (0.97 to 1.47)
1 year	1.16 (0.91 to 1.47)	1.28 (1.05 to 1.57)
2 years	1.16 (0.94 to 1.43)	1.38 (1.14 to 1.67)
Total translation ^b^		
3 months	0.36 (0.29 to 0.46)	0.42 (0.33 to 0.54)
6 months	0.40 (0.30 to 0.53)	0.52 (0.41 to 0.66)
1 year	0.47 (0.38 to 0.58)	0.52 (0.41 to 0.66)
2 years	0.41 (0.32 to 0.51)	0.51 (0.38 to 0.68)
Total rotation^b^		
3 months	1.31 (1.01 to 1.70)	1.23 (0.99 to 1.52)
6 months	1.37 (0.97 to 1.95)	1.40 (1.11 to 1.77)
1 year	1.50 (1.10 to 2.05)	1.55 (1.24 to 1.94)
2 years	1.60 (1.23 to 2.09)	1.69 (1.42 to 2.01)

**^a^**Statistically significant difference between P and PBM.

**^b^**2-sample Mann–Whitney U-test.

### Radiographic results

Radiographic evaluation revealed a mean inclination of 42°(CI 40–43) and mean anteversion of 20° (CI 18–21) with no statistically significant difference between groups (p > 0.3). Only 1 patient (PBM) had a radiolucent line of more than 1 mm (1.03 mm) in zone 3 evaluated on 2-year radiographs. This patient had an early migration of the cup of 0.3 mm medially and 0.3 mm proximally at 3-month follow-up, and stabilized hereafter.

### Patient-reported and clinical outcomes

**Figure 6.**
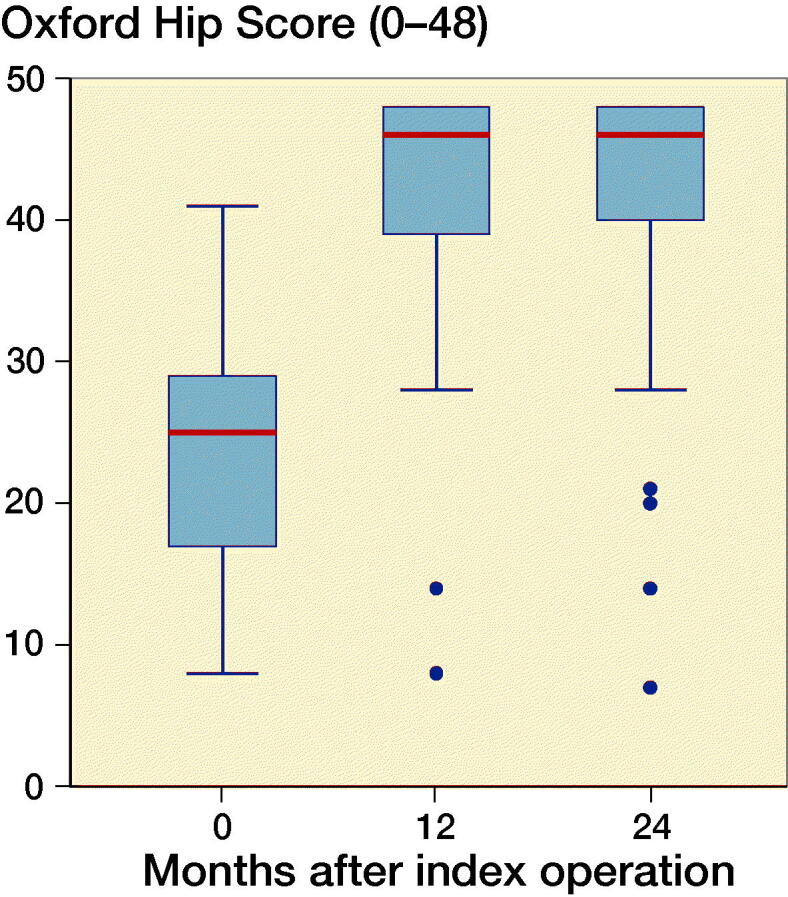
Oxford Hip Score in a box plot. The line tags the median, the box tags the interquartile range (IQR). Whiskers indicate the most extreme value within upper/lower quartile ±1.5ЧIQR.

At 2-year follow-up OHS reached a median value of 46 points (7–48). OHS showed clinically relevant improvement in self-perceived hip function for both groups from baseline to 2-year follow-up of 17 points (CI 14–20) with no statistically significant difference between groups (p = 0.1). Similarly, HOOS increased significantly in all groups from baseline to 2-year follow-up (p < 0.001). PBM-coated cups had a greater increase in HOOS symptoms score than P-coated cups (p = 0.04). However, the difference of 14 points (CI 27–50) was not considered clinically relevant (Paulsen et al. 2014). At 1-year and 2-year follow-up, there were no statistically significant differences between the groups in any HOOS category or OHS (p > 0.5) (Figures 5 and 6).

Figure 5.Median HOOS (0–100).

**Figure:**
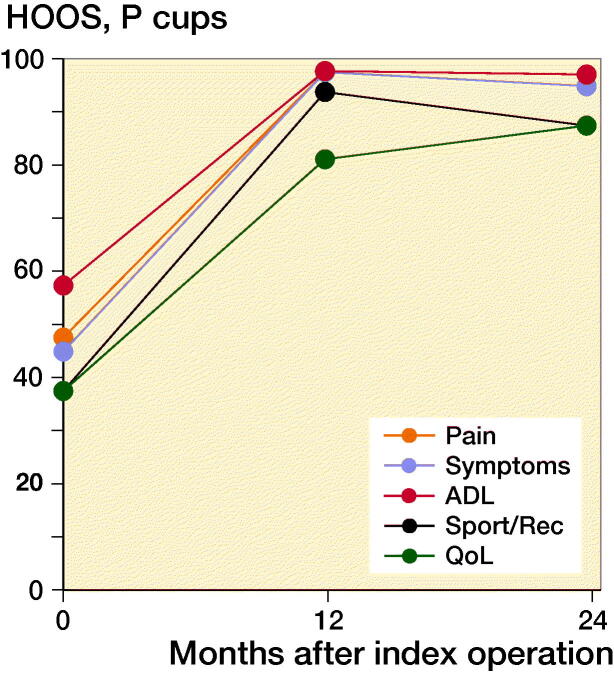

**Figure:**
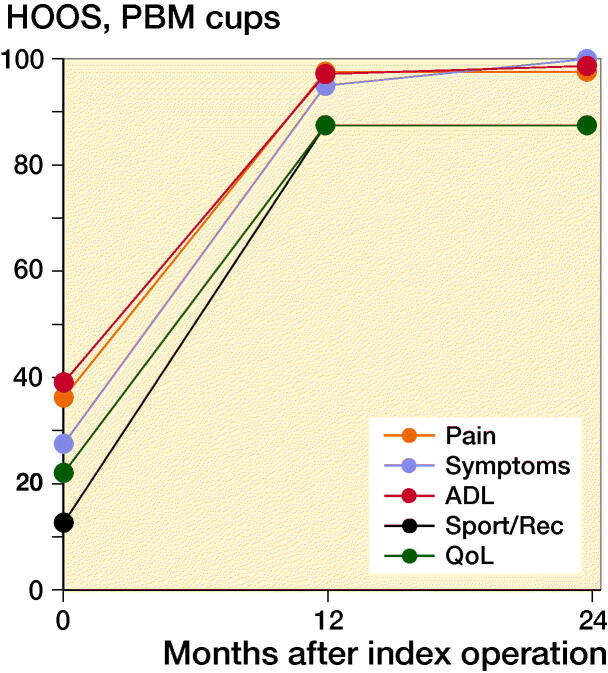

A clinical evaluation at 2-year follow-up comprised 2 head/liner replacements related to dislocation and instability (1 P and 1 PBM).

## Discussion

This study evaluates the migration of porous Exceed cups with or without BM coating, and we found that the PBM-coated cups had higher proximal migration at 6-month follow-up, but there was no statistically significant difference between groups at 2-year follow-up.

### RSA

Building on the experimental results of BM we expected better early bone ingrowth and consequently better fixation in the bone/implant interface in the PBM group (Schmidmaier et al. 2002, Daugaard et al. 2010). Clinically we found that PBM-coated cups had a higher proximal migration at 6-month follow-up, but no statistically significant difference in comparison with P-coated cups was found at 2-year follow-up.

An earlier study on dogs showed that electrochemically applied HA coating was not visible after 4 weeks (Daugaard et al. 2010). This suggests that the coating has been resorbed leaving a space between the implant and bone. Such a gap may explain the increased migration of the PBM implant during the earliest months in the present study.

To our knowledge, this is the first randomized clinical study of electrochemically applied HA coating on cups. Lazarinis et al. (2014) studied a cohort of hemispherical trabeculae-oriented-pattern cups with electrochemically applied HA. Similar to our results, they found an initial proximal migration that seemed to stabilize after 3 months.

We also found statistically significant difference in the y-rotation (anteversion/retroversion) at 3-month follow-up. However, y-rotation varies over time and this finding is likely to be caused by variation.

In accordance with studies on plasma-sprayed HA coatings we found little or no positive effect of electrochemically applied HA coating on migration of hemispherical cups after 2 years (Rohrl et al. 2004, Valancius et al. 2013). Our findings also indicate no improved long-term survival effect of electrochemically applied HA coating. This is in agreement with recent larger registry-based analysis and meta-analysis of HA coatings on cementless cups (Chen et al. 2015, Lazarinis et al. 2017).

Both the P-coated cups and the PBM-coated cups stabilized after 6 months, and early RSA-measured cup stabilization is in agreement with a number of other studies of cementless hemispheric cups (Lazarinis et al. 2014, Salemyr et al. 2015, Hjorth et al. 2017, Nilsson et al. 2017).

Only 1 other clinical study on BoneMaster coating exists in the literature, and it concerns findings of increased early retroversion of femoral stems coated with BM compared with plasma-sprayed HA (Flatoy et al. 2016). In agreement with our results, their femoral stems all stabilized after 3-month follow-up.

### BMD

Low systemic bone quality (T-score < –1.0) has been shown to increase proximal cup migration at 3- and 6-month follow-up (Finnila et al. 2016). In order to avoid migration bias from patients with poor bone quality we excluded all patients with osteoporosis (T-score < –2.5) based on a preoperative DEXA scan of systemic BMD. In spite of randomization, there was a difference in distribution of BMD between the 2 groups. This may explain some of the early difference in proximal cup migration.

### Precision

We included 4 patients with a CN between 150 and 200 on RSA analysis, and upgraded the RSA system during the study. However, the precision of proximal migration was comparable to similar studies on hemispheric cups (Hjorth et al. 2017, Shareghi et al. 2017).

### Patient-reported outcome

In general, patients receiving P cups had a better self-perceived hip function at baseline compared with the PBM group. Although preoperative score does not affect the Patient Acceptable Symptom State (PASS) postoperatively it may explain why PBM cups had superior improvement from baseline to 2-year follow-up in HOOS Symptoms sub-score (Paulsen et al. 2014). Postoperatively, patients reached OHS exceeding 40 points, which is the threshold corresponding to PASS after THR, and also corresponding to the Danish background population-based value of OHS (Paulsen et al. 2012, Keurentjes et al. 2014).

### Strengths and weaknesses

This study sample size calculation was originally based on group mean comparisons of cup migration in general after 5 years. After initiation of this study, Pijls et al. (2012) published a paper validating 2-year follow-up of proximal migration (Y-axis) as proxy measure for later revision. Therefore, we focused the primary endpoint of this study on proximal cup migration after 2 years, and reduced multiple testing of outcomes. The decision to change endpoint was made without knowledge of actual data as described by Evans (2007).

The randomized blinded design and high precision of RSA are major strengths, along with a high degree of patient compliance with almost full follow-up. The generalizability is limited to patients within the study criteria, which were many, because we wanted to make groups as comparable as possible in order to limit variation and be able to find even small differences between coating groups. Longer-term effects of BoneMaster on polyethylene wear cannot be evaluated before 5 years’ follow-up because of the low wear in highly crosslinked UHMWPE liners.

### Summary

We found a higher early proximal cup migration with porous and BoneMaster coating as compared with porous coating alone, which indicates no relevant positive effect of electrochemically applied HA (BoneMaster) on cup osseointegration. Based on this study and the current literature, we see no advantage in additional BoneMaster coating on porous-coated acetabular cups.

### Supplementary data

Table 2 is available as supplementary data in the online version of this article, http://dx.doi.org/10.1080/17453674.2019.1687860

## Supplementary Material

Supplemental Material
