# Supplementary material for: Higher early proximal migration of hemispherical cups with electrochemically applied hydroxyapatite (BoneMaster) on a porous surface compared with porous surface alone: a randomized RSA study with 53 patients
Source: Acta Orthop. 2019 Nov 8;91(1):26–32. doi: 10.1080/17453674.2019.1687860 (PMC7008237; doi:10.1080/17453674.2019.1687860)
Supplement: Supplemental Material [file IORT_A_1687860_SM9840.pdf]

Supplementary data

Table 2. Precision of RSA

	Translation (mm) (n = 49)			Rotation (°) (n = 49)		
	X-axis	Y-axis	Z-axis	X-axis	Y-axis	Z-axis
Mean difference	0.03	-0.01	0.00	–0.12	–0.45	0.10
SD diff.	0.18	0.11	0.31	0.80	0.76	0.36
CR	0.35	0.21	0.61	1.56	1.49	0.36
LoA						
upper limit	0.38	0.20	0.61	1.45	1.04	0.80
lower limit	–0.32	–0.22	–0.61	–1.68	–1.94	–0.60

Mean diff: The systematic difference of RSA.
SD diff: Random variation of RSA.
CR: Coefficient of repeatability (SD×1.96). Indicates the RSA precision for individual recordings.
LoA: Limits of agreement/prediction interval.
